# Rotating Night Shift Work and Healthy Aging After 24 Years of Follow-up in the Nurses' Health Study

**DOI:** 10.1001/jamanetworkopen.2022.10450

**Published:** 2022-05-04

**Authors:** Hongying Shi, Tianyi Huang, Eva S. Schernhammer, Qi Sun, Molin Wang

**Affiliations:** 1 Department of Epidemiology and Health Statistics, School of Public Health and Management, Wenzhou Medical University, Zhejiang, China; 2 Department of Epidemiology, Harvard T. H. Chan School of Public Health, Boston, Massachusetts; 3 Channing Division of Network Medicine, Department of Medicine, Brigham and Women's Hospital, Boston, Massachusetts; 4 Department of Nutrition, Harvard T. H. Chan School of Public Health, Boston, Massachusetts; 5 Department of Epidemiology, Center for Public Health, Vienna, Austria; 6 Department of Biostatistics, Harvard T. H. Chan School of Public Health, Boston, Massachusetts

## Abstract

**Importance:**

Rotating night shift work is associated with higher mortality. Whether it is also associated with overall health among those who survive to older ages remains unclear.

**Objective:**

To examine whether rotating night shift work is associated with healthy aging after 24 years of follow-up in the Nurses’ Health Study, a cohort study among registered female nurses.

**Design, Setting, and Participants:**

For this cohort study, a composite healthy aging phenotype was ascertained among 46 318 participants who were aged 46 to 68 years and free of major chronic diseases in 1988 when the history of night shift work was assessed. In a secondary analysis in which cognitive function decline was considered in the healthy aging definition, 14 273 nurses were involved. Data were analyzed from March 1 to September 30, 2021.

**Exposures:**

Duration of rotating night shift work.

**Main Outcomes and Measures:**

Healthy aging was defined as reaching at least 70 years of age and being free of 11 major chronic diseases, memory impairment, physical limitation, or deteriorated mental health.

**Results:**

Of 46 318 female nurses (mean [SD] age at baseline, 55.4 [6.1] years), 3695 (8.0%) achieved healthy aging after 24 years of follow-up. After adjusting for established and potential confounders, compared with women who never worked rotating night shifts, the odds of achieving healthy aging decreased significantly with increasing duration of night shift work. The odds ratios were 0.96 (95% CI, 0.89-1.03) for 1 to 5 years, 0.92 (95% CI, 0.79-1.07) for 6 to 9 years, and 0.79 (95% CI, 0.69-0.91) for 10 or more years of night shift work (*P* = .001 for trend). This association did not differ substantially by age and lifestyles and was consistent for 4 individual dimensions of healthy aging. Results were similar in a secondary analysis, with an odds ratio of 0.73 (95% CI, 0.60-0.89; *P* < .001 for trend) comparing 10 or more years of night shift work vs no night shift work.

**Conclusions and Relevance:**

In this cohort study, rotating night shift work was associated with decreased probability of healthy aging among US female nurses. These data support the notion that excess night shift work is a significant health concern that may also lead to deteriorated overall health among older individuals.

## Key Points

**Question:**

Is rotating night shift work prospectively associated with healthy aging that simultaneously considers chronic diseases, cognitive and physical function, and mental health?

**Findings:**

In a large cohort study of 46 318 female nurses, long-term rotating night shift work was associated with modestly decreased odds of healthy aging after 24 years of follow-up.

**Meaning:**

In addition to the existing literature suggesting that shift work is associated with increased mortality, the findings of this study further suggest that shift work is also associated with worse overall health among women who survive to older ages.

## Teaser

This cohort study of female nurses examines the association between rotating night shift work and healthy aging after 24 years of follow-up in the Nurses’ Health Study.

## Introduction

Night shift work is becoming increasingly common worldwide, and 15% to 33% of the working population^1,2,3^ are engaged in night shift work, especially among health care workers.^2^ Previous studies have suggested that night shift work may result in circadian disruption, sleep disturbances, and other behavioral changes,^4^ leading to increased risk of chronic diseases,^4,5,6,7,8,9,10,11^ mental disorders,^1,12,13^ cognition impairment,^14,15^ and mortality.^14,16^ The World Health Organization’s International Agency for Research on Cancer concludes that shift work is probably carcinogenic for humans.^17,18^

However, although circadian rhythms are considered a universal and fundamental mechanism in essentially every physiologic process across the entire human body,^19,20^ existing studies^7,8,9,10,11,13,15^ on rotating night shift work have primarily focused on individual health outcomes, and less is known regarding its association with overall health. Particularly, given the interplay between aging and the circadian clock,^19,21^ it is critical to understand the association between night shift work and overall health in older populations. As the aging population increases in the US and other countries, it is critical to identify and examine modifiable factors, such as night shift work, that are potentially associated with the overall health status among older individuals. Depending on the definition of healthy aging,^22,23,24^ an estimated one-third of the population older than 60 years can be considered as healthy agers. In this prospective study, we leveraged the longitudinal follow-up data from the Nurses’ Health Study to examine the association of duration of rotating night shift work with heathy aging (as measured by a full spectrum of health outcomes) among women.

## Methods

### Study Design and Participants

The Nurses’ Health Study is a prospective cohort study of 121 701 US registered nurses aged 30 to 55 years that was established in 1976. Follow-up questionnaires have been used to update the information every 2 years. The cumulative follow-up rate was greater than 90% in this cohort.^25^ Women were asked to report their history of rotating night shift work in 1988, which was treated as baseline in the current cohort study. In our primary analysis, the end of follow-up was through 2012,^26^ when the overall health status, including chronic diseases, physical function, mental health, and memory function, was evaluated (Figure). Of 90 042 women who answered the 1988 questionnaire and were 46 years or older in 1988 (ie, could reach 70 years of age in 2012), we excluded those who had any of 11 main chronic diseases at baseline (n = 17 872), had missing information on rotating night shift work (n = 13 552), or had missing data on healthy aging phenotype in 2012 (n = 12 300), leaving 46 318 women (age range, 46-68 years) in our primary analyses. Participants excluded for missing information on healthy aging did not differ substantially from those included with regard to distributions of major potential confounders (eTable 1 in the Supplement). In our secondary analysis, healthy aging phenotype was defined among 19 415 women who completed a cognitive function test when they reached 70 years of age in 1995 to 2000.^25^ After excluding women with missing information on rotating night shift work history, any of 11 main chronic diseases at baseline, or healthy aging phenotype, 14 273 women were included (eFigure in the Supplement). The study protocol was approved by the institutional review boards of the Brigham and Women’s Hospital and the Harvard T. H. Chan School of Public Health. Participants provided implied consent by returning the questionnaires. This study followed the Strengthening the Reporting of Observational Studies in Epidemiology (STROBE) reporting guideline.

**Figure.**
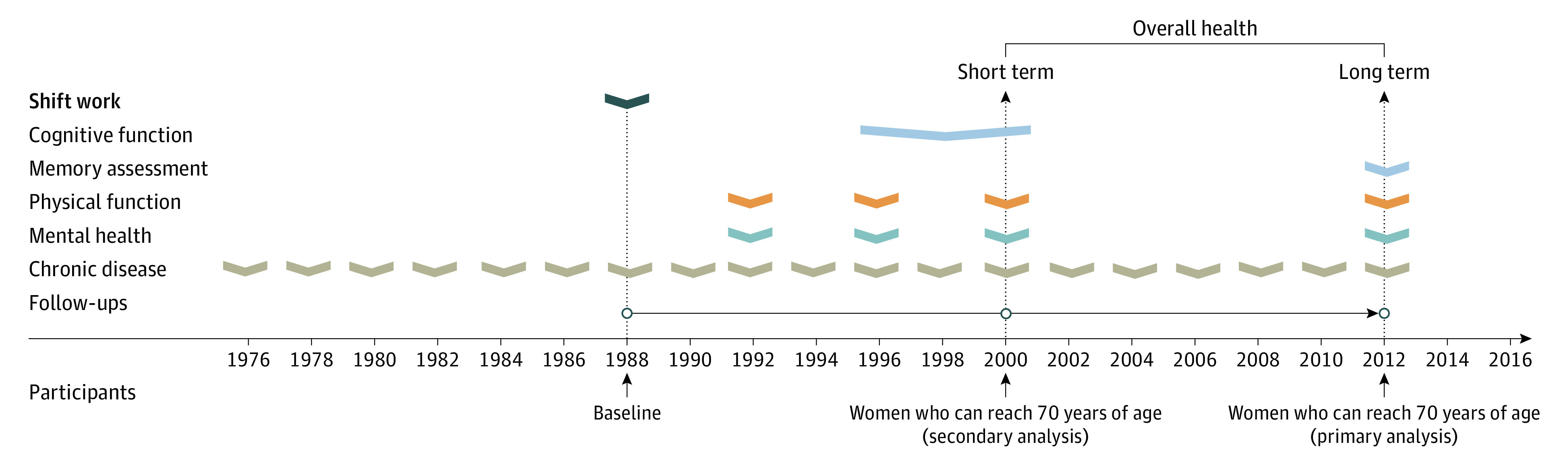
Study Design for Rotating Night Shift Work and Healthy Aging in the Nurses’ Health Study Participants who were free of major chronic diseases in 1988 were followed up for 24 years in the primary analysis (1988-2012; n = 46 319; age range at baseline, 46-68 years) and for 12 years in the secondary analysis (1988-2000; n = 14 273; age range at baseline, 58-68 years).

### Assessment of Healthy Aging

On the basis of the concept of successful aging developed by Rowe and Kahn^27^ and previous literature,^25,26,28,29^ healthy aging was defined as survival to at least 70 years of age and 4 health domains (no major chronic diseases and no impairment in cognitive function, physical function, or mental health). Participants who did not meet any of these criteria were defined as usual agers. Detailed descriptions of our primary outcome, secondary outcome, and each of the 4 domains are listed in Table 1.^30,31,32,33,34,35,36,37,38^ Briefly, in the primary analysis, the outcome was evaluated in 2012 based on 46 318 nurses' data on disease (eMethods 1 in the Supplement), physical function, mental health, and memory function, whereas in the secondary analysis, it was evaluated in 1995 to 2000 based on 14 273 nurses' data on disease, physical function, mental health, and cognitive function.

**Table 1.** Definition and Dimensions of Healthy Aging

Dimension	Primary analysis: healthy aging in 2012	Secondary analysis: healthy aging in 1995-2000
Assessment of chronic diseases	Clinical diagnoses of 11 major chronic diseases^a^ were queried on each biennial questionnaire since 1988, which were then confirmed by professional staff through medical record or pathology report review, telephone interview, or supplementary questionnaire inquiries, and has been shown to be valid in this cohort.^25^ Women who reported no history of these diseases before the end of follow-up (2000 for short-term and 2012 for long-term follow-up) were considered to be free of chronic diseases.	
Assessment of cognitive (or memory) function	In 2012, subjective memory concerns were assessed through the Structured Telephone Interview for Dementia Assessment using 7 questions, which were associated with objective cognitive function in this cohort^30^ and have previously been used to detect individuals with possible cognitive impairment.^31^ Research in other cohorts has established that subjective memory correlates well with dementia.^32^ No impairment in memory was defined as at most 1 memory concern.^26,33^	Between 1995 and 2000, we administered the TICS, a telephone version of the MMSE. TICS scores range from 0 to 41, and a score <31 is considered cognitive impairment.^34^ Studies have shown high test-retest reliability and validity of TICS in assessing cognitive status. A strong correlation (*r* = 0.97) was found between TICS and MMSE assessments.^35^ Trained study nurses who were unaware of the study hypothesis performed the assessment, and inter-interviewer reliability was high (>0.95).^36^
Assessment of physical function	In 2012, physical function was assessed by 10 questions^b^ in the SF-36. Impairment of physical function was defined as any of the following: limited at least a little on moderate activities or limited a lot on more difficult physical tasks; otherwise, they were defined as no impairment of physical function.^26^	In 1996, 1998, and 2000 (the one nearest to cognitive assessment was chosen), physical function was assessed by the same question as in 2012.
Assessment of mental health	In 2012, mental health was assessed using the GDS-15) (range, 0-15, with lower scores indicating better mental health).^37^ Good mental health was defined as a GDS-15 score ≤1 (the median value in the cohort).	In 1996, 1998, and 2000 (the one nearest to cognitive assessment was chosen), mental health was evaluated by 5 questions^c^ in the SF-36. A score between 1 (worst) and 6 (best) was assigned to each question. We then summed these scores and rescaled them on a range of 0-100. Good mental health was defined as a score >84 (the median value in this cohort).^25^
Healthy aging	Healthy aging was defined as surviving to the end of follow-up (age ≥70 y), being free of major chronic diseases, and having no impairment of physical function, no mental health limitations, and no impairment of cognition (in 1995-2000) or subjective memory (in 2012).^25,26^ Participants who did not meet any of these criteria were defined as usual agers.	

Abbreviations: GDS-15, Geriatric Depression Scale 15; MMSE, Mini-Mental State Examination; SF-36, Medical Outcomes Study Short-Form Health Survey; TICS, Telephone Interview of Cognitive Status.

^a^ Major chronic diseases covers most common conditions that would significantly deteriorate human health, including cancer (except for nonmelanoma skin cancers), diabetes, myocardial infarction, coronary artery bypass graft surgery or percutaneous transluminal coronary angioplasty (as a surrogate for coronary artery disease), congestive heart failure, stroke, kidney failure, chronic obstructive pulmonary disease, Parkinson disease, multiple sclerosis, and amyotrophic lateral sclerosis.

^b^ The 10 questions inquired about physical limitations in performing the following activities: moderate activities (eg, moving a table, pushing a vacuum cleaner, bowling, or playing golf); bathing and dressing yourself; walking 1 block; walking several blocks; walking more than 1 mile; vigorous activities (eg, running, lifting heavy objects, or strenuous sports); bending, kneeling, or stooping; climbing 1 flight of stairs; climbing several flights of stairs; and lifting or carrying groceries. Each question had 3 response choices: “Yes, limited a lot,” “Yes, limited a little,” or “No, not limited at all.” The validity and reproducibility of the SF-36 and its components have been previously established.^38^

^c^ The 5 questions were as follows: Have you been a very nervous person? Have you felt so down in the dumps nothing could cheer you up? Have you felt calm and peaceful? Have you felt downhearted and blue? and Have you been a happy person? There were 6 possible responses to each question ranging from none of the time to all of the time.

### Assessment of Rotating Night Shift Work

In 1988, women were asked to report their total number of years of rotating night shift work (defined as at least 3 nights per month in addition to day and evening shifts) with 8 prespecified categories, including never, 1 to 2 years, 3 to 5 years, 6 to 9 years, 10 to 14 years, 15 to 19 years, 20 to 29 years, and 30 years or more. Duration of rotating night shift work has previously been associated with risk of diabetes,^8^ coronary heart disease,^7^ and breast cancer^39^ in this cohort. Consistent with previous publications,^7,8^ we categorized the duration of rotating night shift work into 4 categories: never, 1 to 5 years, 6 to 9 years, and 10 years or more.

### Assessment of Covariates

Information on a broad range of covariates was obtained, including demographic characteristics,^40^ such as marital status, race, educational level, and household income; lifestyle factors, such as dietary data,^41,42,43^ sleep behavior, and physical activity^44,45,46^ (eMethods 2 in the Supplement); family history of cancer, myocardial infarction, and diabetes; clinical diagnoses of hypertension and high cholesterol; use of supplemental multivitamins and aspirin; and menopausal status and postmenopausal hormone use.

### Statistical Analysis

Logistic regression models were used to estimate odds ratios (ORs) and 95% CIs for healthy aging across rotating night shift work categories (none, 1-5 years, 6-9 years, and ≥10 years). Women with no history of rotating night shift work were set as the reference category. An OR smaller than 1 indicates decreased odds of healthy aging. In the base model, we adjusted for age, educational level, marital status, household income, hypertension, high cholesterol, family history (cancer, myocardial infarction, or diabetes), menopausal status and hormone use, and aspirin use. In the multivariate-adjusted model, we additionally adjusted for lifestyle factors, including smoking history, alcohol intake, total energy intake, diet quality (Alternate-Healthy Eating Index), physical activity, standing and sitting time per day, and daily sleep duration. Obesity may be a consequence of night shift work,^47,48^ and obesity had a strong inverse association with healthy aging.^49^ By inference, obesity may act as a mediator between night shift work and healthy aging; therefore, we further adjusted for body mass index (BMI; calculated as weight in kilograms divided by height in meters squared) in a separate model. Mediation analysis based on the counterfactual framework^50^ was also performed. Dummy variables were created to indicate missing covariate values. A *P* value for trend was obtained by assigning the midpoint value to each rotating night shift work category and modeling it as a continuous variable.

We performed several sensitivity analyses to examine the robustness of observed associations. First, to further control for potential selection bias, we performed a propensity-weighted analysis. In this analysis, the exposure was treated as a binary variable (those who worked the night shift vs those who did not). Second, we restricted our analyses to women without hypertension or hypercholesterolemia. Third, we evaluated potential changes in the association estimates if we additionally adjusted for snoring, waist-hip ratio, or coffee intake or removed sleep duration from the multivariate model (because sleep duration could be an intermediate between rotating night shift work and healthy aging). In addition, to explore the possible influence of age and lifestyle factors^51^ on the association, we performed stratified analysis (eMethods 3 in the Supplement). Finally, to evaluate the association between years of night shifts and healthy aging among survivors, we excluded women who died before the end of follow-up from the group of usual agers and repeated all analyses.

Data were analyzed from March 1 to September 30, 2021. All analyses were performed with SAS software, version 9.4 (SAS Institute Inc). A 2-sided *P* < .05 was considered statistically significant.

## Results

Of the 46 318 female nurses included in the primary analysis, 17 786 (38.4%) remained free of the 11 chronic diseases, 7150 (15.4%) had no impairment of physical function, 19 654 (42.4%) had good mental health, and 23 169 (50.0%) reported no impairment of memory function. A total of 3695 participants (8.0%) met all criteria of healthy aging; the rest were usual agers.

### Baseline Characteristics of Participants

The mean (SD) age of study participants at baseline was 55.4 (6.1) years. A total of 45 300 participants (97.8%) were White, 562 (1.2%) were Black, 98 (0.2%) were American Indian, 347 (0.8%) were Asian, and 11 (0.02%) were Hawaiian. Of the 46 318 women studied, 27 480 (59.3%) reported having ever engaged in rotating night shift work, and 5384 (11.6%) reported at least 10 years of rotating night shift work. Table 2 gives the baseline characteristics of the study participants across categories of duration of rotating night shift work. Compared with women with no history of rotating night shift work, those with more years of rotating night shifts were slightly older (mean [SD] age, 56.7 [6.0] years for those with ≥10 years of shift work vs 55.1 [6.1] for those with no shift work), had less education (master’s or doctorate degree, 341 [7.1%] for those with ≥10 years of shift work vs 1880 [10.8%] for those with no shift work), slept somewhat less (6.8 [1.1] hours per day for those with ≥10 years of shift work vs 7.0 [1.0] hours per day for those with no shift work), were more likely to be current smokers (1347 [25.0%] for those with ≥10 years of shift work vs 3241 [17.2%] for those with no shift work) or regular snorers (538 [11.5%] for those with ≥10 years of shift work vs 1451 [8.7%] for those with no shift work), had higher mean (SD) BMIs (26.4 [5.2] for those with ≥10 years of shift work vs 25.1 [4.5] for those with no shift work), had less median (IQR) sitting time (2.2 [1.1-4.4] hours per day for those with ≥10 years of shift work vs 4.4 [1.1-4.4] hours per day for those with no shift work), and were more likely to have hypertension (983 [18.3%] for those with ≥10 years of shift work vs 2940 [15.6%] for those with no shift work).

**Table 2.** Age-Adjusted Baseline Characteristics in 1988 by Years of Rotating Night Shift Work in the Nurses’ Health Study^a^

Characteristic	Duration of night shift work			
	Never (n = 18 838)	1-5 y (n = 18 944)	6-9 y (n = 3152)	≥10 y (n = 5384)
Length of rotating night shift work, median (IQR), y	0	1.5 (1.5-4.0)	7.5 (7.5-7.5)	17.0 (12.0-24.5)
Age in 1988,^b^ mean (SD) [range], y	55.1 (6.1) [46-68]	55.3 (6) [46-68]	55.9 (6) [46-68]	56.7 (6) [46-68]
Education, No. (%)				
Registered nurse	11 854 (67.9)	12 146 (68.9)	2061 (71.2)	3684 (76.2)
Bachelor’s degree	3712 (21.3)	3584 (20.3)	544 (18.8)	808 (16.7)
Master’s or doctorate degree	1880 (10.8)	1893 (10.7)	288 (10.0)	341 (7.1)
Husband’s educational level, No. (%)				
High school or less	6710 (44.9)	6429 (42.7)	1175 (48.2)	2271 (56.4)
College graduate	4466 (29.9)	4519 (30.0)	714 (29.3)	1083 (26.9)
Graduate school	3758 (25.2)	4123 (27.4)	548 (22.5)	675 (16.8)
Self-reported race, No. (%)				
American Indian	30 (0.2)	43 (0.2)	5 (0.2)	20 (0.4)
Asian	155 (0.8)	131 (0.7)	22 (0.7)	39 (0.7)
Black	198 (1.1)	222 (1.2)	45 (1.4)	97 (1.8)
Hawaiian	5 (0.03)	3 (0.02)	2 (0.06)	1 (0.02)
White	18 450 (97.9)	18 545 (97.9)	3078 (97.7)	5227 (97.1)
Marital status, No. (%)				
Married	16 082 (92.7)	16 289 (92.9)	2607 (89.8)	4443 (89.9)
Widowed	489 (2.8)	458 (2.6)	97 (3.3)	189 (3.8)
Separated‚ divorced‚ or never married	776 (4.5)	780 (4.5)	199 (6.8)	312 (6.3)
Family annual income in 1986, median (IQR), $ in 10 000s	6.0 (4.7-7.8)	6.1 (4.7-7.9)	5.8 (4.6-7.5)	5.6 (4.5-7.2)
BMI at baseline, mean (SD)	25.1 (4.5)	25.2 (4.5)	25.7 (4.8)	26.4 (5.2)
Smoking status, No. (%)				
Never smoker	8573 (45.5)	8324 (43.9)	1305 (41.4)	2236 (41.5)
Past smoker	7024 (37.3)	7335 (38.7)	1167 (37.0)	1801 (33.5)
Current smoker	3241 (17.2)	3285 (17.3)	679 (21.6)	1347 (25.0)
Alcohol intake, No. (%), g/d				
None	7173 (40.1)	6942 (38.5)	1194 (40.2)	2239 (44.1)
1-14.9	8362 (46.7)	8637 (47.8)	1406 (47.3)	2289 (45.1)
≥15	2356 (13.2)	2475 (13.7)	373 (12.5)	547 (10.8)
AHEI, mean (SD)	47.4 (10.9)	47.8 (10.8)	47.9 (10.7)	47.1 (10.6)
Total calories, mean (SD), kcal	1734.2 (519.8)	1774.6 (527.3)	1759.8 (539.0)	1772.8 (553.4)
Total coffee, median (IQR), cups per day	2.5 (1.0-3.5)	2.5 (1.0-3.5)	2.5 (1.0-3.5)	2.5 (1.0-4.5)
Physical activity, median (IQR), MET-h/wk^c^	8.4 (3.1-20.2)	9.7 (3.6-21.2)	9.3 (3.5-21.5)	8.6 (3.2-21.5)
Time, median (IQR), h/d				
Standing	4.4 (1.6-7.2)	4.4 (1.6-7.2)	4.4 (1.6-7.2)	4.4 (1.6-7.7)
Sitting	4.4 (1.1-4.4)	4.4 (1.1-4.4)	2.2 (1.1-4.4)	2.2 (1.1-4.4)
Family history, No. (%)				
Diabetes	5230 (27.8)	5454 (28.8)	990 (31.4)	1677 (31.1)
Myocardial infarction	3421 (18.2)	3515 (18.6)	613 (19.5)	1074 (20.0)
Cancer	2656 (14.1)	2782 (14.7)	451 (14.3)	737 (13.7)
History, No. (%)				
Hypertension	2940 (15.6)	2926 (15.4)	554 (17.6)	983 (18.3)
High cholesterol	3606 (19.1)	3624 (19.1)	583 (18.5)	1010 (18.8)
Use of multivitamin, No. (%)	7176 (38.6)	7429 (39.7)	1197 (38.6)	2097 (39.6)
Menopausal status and hormone use, No. (%)				
Premenopausal	4079 (22.3)	4106 (22.3)	681 (22.3)	1027 (19.7)
Postmenopausal and never used	8657 (47.4)	8637 (46.9)	1503 (49.3)	2792 (53.4)
Postmenopausal and past user	560 (3.1)	553 (3.0)	98 (3.2)	145 (2.8)
Postmenopausal and current user	4970 (27.2)	5116 (27.8)	767 (25.1)	1263 (24.2)
Regular aspirin use (≥2 tablets per week), No. (%)	5837 (32.7)	5988 (33.2)	1007 (33.4)	1804 (35.2)
Sleep duration, mean (SD), h/d	7.0 (1.0)	7.0 (1.0)	6.9 (1.0)	6.8 (1.1)
Regular snorer, No. (%)	1451 (8.7)	1485 (8.8)	292 (10.5)	538 (11.5)

Abbreviations: AHEI, Alternate-Healthy Eating Index; BMI, body mass index (calculated as weight in kilograms divided by height in meters squared); MET, metabolic equivalent.

^a^ Data are standardized to the age distribution of the study population.

^b^ Value was not age adjusted.

^c^ MET-h/wk is calculated as the sum of the mean time per week spent in each activity × MET value of each activity.

### Primary Analysis: Duration of Rotating Night Shift Work and Healthy Aging in 2012

Table 3 summarizes the ORs of healthy aging in 2012 associated with rotating night shift work. Compared with women without rotating night shift work, the multivariate-adjusted ORs were 0.96 (95% CI, 0.89-1.03) for women with 1-5 years of rotating night shift work, 0.92 (95% CI, 0.79-1.07) for women with 6-9 years of rotating night shift work, and 0.79 (95% CI, 0.69-0.91) for those with 10 years or more of rotating night shift work (*P* = .001 for trend). Additional adjustment for BMI attenuated the association (adjusted OR, 0.97; 95% CI, 0.90-1.05 for 1-5 years; adjusted OR, 0.98; 95% CI, 0.84-1.15) for 6-9 years; and adjusted OR, 0.88; 95% CI, 0.77-1.01 for ≥10 years; *P* = .11 for trend). This finding was further confirmed in the formal mediation analysis (eTable 2 in the Supplement): 42.9% to 61.6% of the association was mediated by overweight or BMI in 1988, and the natural direct effects were statistically nonsignificant.

**Table 3.** Odds Ratios (95% CIs) of Healthy Aging by History of Rotating Night Shift Work in the Nurses’ Health Study (1988-2012)

Outcome	No.	Healthy aging, No. (%)	Base adjusted^a^	Multivariate adjusted^b^	Multivariate and BMI adjusted^c^
Healthy aging by duration of shift work, y					
0	18 838	1653 (8.8)	1 [Reference]	1 [Reference]	1 [Reference]
1-5	18 944	1547 (8.2)	0.97 (0.90-1.05)	0.96 (0.89-1.03)	0.97 (0.90-1.05)
6-9	3152	218 (6.9)	0.92 (0.79-1.07)	0.92 (0.79-1.07)	0.98 (0.84-1.15)
≥10	5384	277 (5.1)	0.78 (0.68-0.90)	0.79 (0.69-0.91)	0.88 (0.77-1.01)
*P* for trend	NA	NA	<.001	.001	.11
Free of main chronic diseases by duration of shift work, y					
0	18 838	7565 (40.2)	1 [Reference]	1 [Reference]	1 [Reference]
1-5	18 944	7528 (39.7)	1.01 (0.96-1.05)	1.01 (0.96-1.05)	1.01 (0.97-1.06)
6-9	3152	1063 (33.7)	0.84 (0.77-0.91)	0.86 (0.79-0.93)	0.88 (0.81-0.96)
≥10	5384	1630 (30.3)	0.78 (0.73-0.84)	0.83 (0.77-0.89)	0.88 (0.82-0.94)
*P* for trend	NA	NA	<.001	<.001	<.001
Good physical function by duration of shift work, y					
0	18 838	3135 (16.6)	1 [Reference]	1 [Reference]	1 [Reference]
1-5	18 944	2996 (15.8)	0.99 (0.93-1.05)	0.97 (0.91-1.03)	0.98 (0.92-1.04)
6-9	3152	431 (13.7)	0.94 (0.84-1.06)	0.94 (0.83-1.06)	1.00 (0.89-1.13)
≥10	5384	588 (10.9)	0.85 (0.77-0.94)	0.87 (0.78-0.96)	0.96 (0.87-1.07)
*P* for trend	NA	NA	.002	.006	.56
Good mental health by duration of shift work, y					
0	18 838	8328 (44.2)	1 [Reference]	1 [Reference]	1 [Reference]
1-5	18 944	8255 (43.6)	1.00 (0.96-1.05)	0.99 (0.95-1.04)	0.99 (0.95-1.04)
6-9	3152	1228 (39.0)	0.91 (0.84-0.99)	0.92 (0.84-0.998)	0.94 (0.86-1.02)
≥10	5384	1843 (34.2)	0.84 (0.78-0.90)	0.87 (0.81-0.93)	0.91 (0.84-0.97)
*P* for trend	NA	NA	<.001	<.001	.003
Good memory function by duration of shift work, y					
0	18 838	9748 (51.8)	1 [Reference]	1 [Reference]	1 [Reference]
1-5	18 944	9628 (50.8)	0.98 (0.94-1.03)	0.98 (0.94-1.03)	0.98 (0.94-1.03)
6-9	3152	1468 (46.6)	0.90 (0.83-0.97)	0.91 (0.84-0.98)	0.91 (0.84-0.99)
≥10	5384	2325 (43.2)	0.88 (0.82-0.94)	0.91 (0.85-0.97)	0.92 (0.86-0.98)
*P* for trend	NA	NA	<.001	<.001	.003

Abbreviations: BMI, body mass index (calculated as weight in kilograms divided by height in meters squared); NA, not applicable.

^a^ Base model adjusted for age at baseline (continuous); educational level (registered nurse, bachelor’s, or master’s or doctorate degree); marital status (married, widowed, or separated/divorced/never married); household income (quintiles); baseline hypertension and high cholesterol (yes or no); family history of cancer, myocardial infarction, and diabetes (yes or no); menopausal status and hormone use (premenopausal, postmenopausal never users, postmenopausal past users, or postmenopausal current users); and aspirin use (regular use or not).

^b^ Multivariate model additionally adjusted for lifestyle factors, including smoking history (never, former smoker, or current smoker), alcohol intake (none, 1-14.9 g/d, or ≥15 g/d), total energy intake (quintiles), diet quality (Alternate-Healthy Eating Index score, in quintiles), physical activity (metabolic equivalent hours per week, in quintiles); standing and sitting time (in quintiles); and sleep duration (≤5, 6, 7, 8, or ≥9 hours).

^c^ Additionally adjusted for BMI at baseline (<18.5, 18.5-24.9, 25-29.9, or ≥30).

Longer years of rotating night shift work were consistently inversely associated with 4 individual dimensions of healthy aging in the multivariate-adjusted model. The multivariate-adjusted ORs comparing women with 10 years or more of rotating night shift work vs women without rotating night shift work were 0.83 (95% CI, 0.77-0.89) for being free of major chronic diseases (*P* < .001 for trend), 0.87 (95% CI, 0.78-0.96) for having good physical function (*P* = .006 for trend), 0.87 (95% CI, 0.81-0.93) for having good mental health (*P* < .001 for trend, and 0.91 (95% CI, 0.85-0.97) for having good memory function (*P* < .001 for trend).

### Secondary Analysis: Duration of Rotating Night Shift Work and Healthy Aging in 1995-2000

Of the 14 273 participants included in the analysis of short-term healthy aging, 8515 women (59.7%) had none of the 11 chronic diseases, 3454 (24.2%) had no impairment of physical function, 5317 (37.3%) had good mental health, and 11 056 (77.5%) reported no impairment of cognitive function. A total of 1386 participants (9.7%) met all criteria of healthy aging in 1995 to 2000; the rest were usual agers.

The associations of rotating night shift work with healthy aging in 1995 to 2000 were consistent with the primary analysis (Table 4). Compared with women without rotating night shift work, the multivariate-adjusted ORs were 1.00 (95% CI, 0.88-1.13) for 1-5 years of shift work, 0.72 (95% CI, 0.56-0.92) for 6-9 years of shift work, and 0.73 (95% CI, 0.60-0.89) for 10 years or more of shift work (*P* < .001 for trend). The association remained essentially unchanged with additional adjustment for BMI (OR, 1.00; 95% CI, 0.89-1.14 for 1-5 years; OR, 0.75; 95% CI, 0.59-0.97 for 6-9 years; and OR, 0.78; 95% CI, 0.64-0.95 for ≥10 years; *P* = .003 for trend). Rotating night shift work was also inversely associated with 4 dimensions of healthy aging. The multivariate-adjusted ORs comparing women with 10 years or more of rotating night shift work vs women without rotating night shift work were 0.84 (95% CI, 0.75-0.93) for being free of major chronic diseases (*P* < .001 for trend), 0.81 (95% CI, 0.71-0.92) for having good physical function (*P* < .001 for trend), 0.92 (95% CI, 0.82-1.03) for having good mental health (*P* = .03 for trend), and 0.89 (95% CI, 0.78-1.00) for having good memory function (*P* = .02 for trend). Additional adjustment for BMI did not change these associations.

**Table 4.** Odds Ratios (95% CIs) of Healthy Aging by History of Rotating Night Shift Work in the Nurses’ Health Study (1988-2000)

Outcome	No.	Healthy aging, No. (%)	Base adjusted^a^	Multivariate adjusted^b^	Multivariate and BMI adjusted^c^
Healthy aging by duration of shift work, y					
0	5477	553 (10.1)	1 [Reference]	1 [Reference]	1 [Reference]
1-5	5722	608 (10.6)	1.03 (0.91-1.17)	1.00 (0.88-1.13)	1.00 (0.89-1.14)
6-9	1044	80 (7.7)	0.75 (0.59-0.97)	0.72 (0.56-0.92)	0.75 (0.59-0.97)
≥10	2030	145 (7.1)	0.75 (0.62-0.91)	0.73 (0.60-0.89)	0.78 (0.64-0.95)
*P* for trend	NA	NA	<.001	<.001	.003
No main chronic diseases by duration of shift work, y					
0	5477	3291 (60.1)	1 [Reference]	1 [Reference]	1 [Reference]
1-5	5722	3563 (62.3)	1.08 (1.00-1.17)	1.07 (0.99-1.16)	1.07 (0.99-1.16)
6-9	1044	572 (54.8)	0.81 (0.71-0.93)	0.80 (0.70-0.92)	0.82 (0.72-0.95)
≥10	2030	1089 (53.7)	0.82 (0.74-0.91)	0.84 (0.75-0.93)	0.87 (0.78-0.97)
*P* for trend	NA	NA	<.001	<.001	<.001
Good physical function by duration of shift work, y					
0	5477	1351 (24.7)	1 [Reference]	1 [Reference]	1 [Reference]
1-5	5722	1474 (25.8)	1.03 (0.94-1.12)	0.99 (0.90-1.08)	0.99 (0.91-1.08)
6-9	1044	216 (20.7)	0.81 (0.69-0.95)	0.76 (0.64-0.90)	0.80 (0.68-0.95)
≥10	2030	413 (20.3)	0.85 (0.75-0.96)	0.81 (0.71-0.92)	0.88 (0.77-1.00)
*P* for trend	NA	NA	.001	<.001	.008
Good mental health by duration of shift work, y					
0	5477	2085 (38.1)	1 [Reference]	1 [Reference]	1 [Reference]
1-5	5722	2181 (38.1)	0.99 (0.92-1.07)	0.98 (0.91-1.06)	0.98 (0.91-1.06)
6-9	1044	356 (34.1)	0.85 (0.74-0.98)	0.85 (0.74-0.98)	0.85 (0.74-0.98)
≥10	2030	695 (34.2)	0.90 (0.80-0.99)	0.92 (0.82-1.03)	0.92 (0.82-1.03)
*P* for trend	NA	NA	.009	.03	.03
Good cognitive function by duration of shift work, y					
0	5477	4266 (77.9)	1 [Reference]	1 [Reference]	1 [Reference]
1-5	5722	4514 (78.9)	1.02 (0.93-1.12)	1.01 (0.92-1.11)	1.01 (0.92-1.11)
6-9	1044	788 (75.5)	0.87 (0.74-1.02)	0.88 (0.75-1.03)	0.88 (0.75-1.04)
≥10	2030	1488 (73.3)	0.86 (0.76-0.97)	0.89 (0.78-1.00)	0.90 (0.79-1.02)
*P* for trend	NA	NA	.003	.02	.03

Abbreviations: BMI, body mass index (calculated as weight in kilograms divided by height in meters squared); NA, not applicable.

^a^ Base model adjusted for age at baseline (continuous); educational level (registered nurse, bachelor’s, or master’s or doctorate degree); marital status (married, widowed, or separated/divorced/never married); household income (quintiles); baseline hypertension and high cholesterol (yes or no); family history of cancer, myocardial infarction, and diabetes (yes or no); menopausal status and hormone use (premenopausal, postmenopausal never users, postmenopausal past users, or postmenopausal current users); and aspirin use (regular use or not).

^b^ Multivariate model additionally adjusted for lifestyle factors, including smoking history (never, former smoker, or current smoker), alcohol intake (none, 1-14.9 g/d, or ≥15 g/d), total energy intake (quintiles), diet quality (Alternate-Healthy Eating Index score, in quintiles), physical activity (metabolic equivalent hours per week, in quintiles); standing and sitting time (in quintiles); and sleep duration (≤5, 6, 7, 8, or ≥9 hours).

^c^ Additionally adjusted for BMI at baseline (<18.5, 18.5-24.9, 25-29.9, or ≥30).

### Sensitivity Analysis

In the propensity-weighted analysis, we found similar associations between rotating night shift work and healthy aging (eTable 3 in the Supplement). We also observed similar results when we restricted our analyses to women who did not report a diagnosis of hypertension or hypercholesterolemia at baseline (eTable 4 in the Supplement). Additional adjustment for snoring, coffee intake, waist-to-hip ratio, or removal of sleep duration from the model did not change these results. In stratified analyses by age and lifestyles, an inverse association between shift work and healthy aging was consistently observed in most of the strata (eTable 5 in the Supplement) except for physical activity. In the analyses excluding 13 352 participants who died during follow-up before 2012 (n = 32 966), the main results remained similar.

## Discussion

In this large, prospective cohort study of US female nurses, duration of rotating night shift work was independently associated with healthy aging after 24 years of follow-up. Ten years or more of rotating night shift work was associated with 20% decreased odds of healthy aging. This association was consistently observed for the individual component of healthy aging. Overall, the observed association did not differ substantially by age, BMI, and other lifestyle factors. Results were similar in a secondary analysis in which memory impairment was replaced with cognitive function decline, with an OR of 0.73 comparing 10 or more years vs no night shift work. To the best of our knowledge, this is the first prospective cohort study to investigate the association of night shift work with overall health status.

### Comparison With Other Studies and Explanations

In addition to the existing literature suggesting that shift work is associated with increased mortality,^14,52,53^ our findings further indicate that this reduced lifespan might also be accompanied by worse overall health and functioning. Our results are also consistent with previous findings on the associations between shift work and individual health conditions. Rotating night shift work has previously been linked to chronic diseases, including cardiovascular disease,^7,9,10,52^ diabetes,^6,54^ cancers,^17,18,55,56^ and mental health,^13,57^ with limited evidence for cognitive function.^58^ However, some previous studies had relatively short^10,54^ or unknown^59^ follow-up duration, prior studies on mental health were mostly cross-sectional,^57^ and the proportion of shift workers was low.^60^ Our study provided more concrete evidence on the association of shift work with overall health by simultaneously considering chronic diseases, cognitive health, and mental and physical function and by evaluating outcomes with extended duration of follow-up.

Although mechanisms have not yet been clearly defined, several potential mechanisms underlie this association. Rotating night shift work alters circadian rhythms, which play important roles in daily metabolic function by regulating patterns of energy expenditure and hormones, such as leptin, ghrelin, thyrotropin, insulin, and melatonin^61^; meanwhile, disruption of circadian rhythms could contribute to insulin resistance, impaired glucose regulation, and development of diseases.^4^ Besides the effect on physical health, persons working night shifts are more likely to experience chronic sleep deprivation, poor-quality sleep, or sleep disorders,^4,62^ which can then lead to disruptions in mental health^1^ and impairment of cognitive function.^63^ A recent study^29^ that used Nurses’ Health Study data observed a nonlinear association between sleep duration and the odds of achieving healthy aging; however, the association between rotating night shift work and healthy aging was not explained by sleep duration in our study. Although long-term shift workers had slightly less sleep duration and were more likely to be regular snorers, our results are similar with or without additional adjustment for sleep duration, in different levels of sleep duration, and among regular or nonregular snorers. In addition, other factors, such as disturbed sociotemporal patterns (resulting from atypical work hours leading to family problems, reduced social support, and stress), might also explain the association.^12^ These findings indicate the potential direct effect of shift work on overall health.

In addition, unfavorable changes in health behaviors (such as obesity) among rotating night shift workers may partly explain the observed association. One meta-analysis^48^ confirmed the risks of developing overweight and obesity associated with night shift work. Overweight or obesity was also a strong independent risk factor of healthy aging.^49^ Our analysis confirmed that overweight or obesity might be an important mediator of the association between shift work and healthy aging. This finding suggests the importance of keeping healthy weight among shift workers, although these results warrant replication.

### Strengths and Limitations

The strengths of this study included the prospective design; use of an integrated, composite measure for healthy aging; long follow-up; large sample size; and detailed information on a wide range of potential confounders. Several limitations of this study should also be considered. This study is limited to rotating night shift work in predominantly White US female nurses. Although the homogeneity of our study participants improved the response rate and the quality of their self-reported health status and minimized confounding by socioeconomic status, shift work patterns may be different by sex, ethnicity, and socioeconomic status.^59^ Therefore, our results may not apply to other populations, and future studies in more diverse samples are needed to confirm our findings. In addition, this is an observational study; therefore, we cannot rule out the possibility of unmeasured residual confounding.

Furthermore, health-related selection (eg, healthy worker effect) could lead to an underestimation of the association between rotating night shift work and healthy aging.^54,64^ It is possible that women who were able to adapt to many years of rotating night shift work were generally healthier than those who only worked on regular daytime schedules or withdrew from work for health reasons. Therefore, the association between rotating night shift work and healthy aging may be underestimated if the reference group included some women who worked on daytime schedules or withdrew from work because of health-related concerns. Another limitation was that we only examined rotating night shift work, a work schedule shown to have the largest impact on sleep, circadian disruption, and chronic disease risk. More research is needed to evaluate the effect of other types of shift work and intensity of shift work on health. In addition, duration of rotating night shift work was only assessed once at baseline, which may lead to misclassification if exposure to night shift work changed after baseline. However, women had a mean age of 55.4 years at baseline, a time when most women no longer performed night shift work and were less likely to start new night shifts.

## Conclusions

In this cohort study, among women who worked as registered nurses, longer duration of rotating night shift work was associated with significantly decreased odds of healthy aging after 24 years of follow-up. This association was also present for each of 4 dimensions of healthy aging. Because an increasing proportion of the working population is involved in rotating night shift work, these findings further highlight the importance of understanding the association of night shift work with human health. Additional studies are warranted to confirm our findings in men and other ethnic populations.

## Notes

Supplement. **eTable 1.** Age-Adjusted Baseline Characteristics by Exclusions **eTable 2.** Mediation of BMI on the Association Between Rotating Night Shift Work and Healthy Aging, NHS (1988-2012) **eTable 3.** Odds Ratios of Healthy Aging by History of Rotating Night Shift Work in the Nurses’ Health Study (1988-2012), Based on Propensity Weighted Analysis, *N* = 46,318 **eTable 4.** Sensitivity Analysis on Rotating Night Shift Work and Healthy Aging in 2012 by Restriction or Adjustment of Additional Potential Confounders, NHS (1988-2012) **eTable 5.** Stratified Analysis for Rotating Night Shift Work and Healthy Aging in 2012, NHS (1988-2012), *N* = 46,318 **eFigure.** Flowchart of Participants’ Selection **eMethods 1.** Confirmation of Different Chronic Diseases **eMethods 2.** Assessment of Covariates **eMethods 3.** Stratified Analysis (Methods and Results)
