# Error in Figure Caption

**DOI:** 10.1001/jamanetworkopen.2022.18151

**Published:** 2022-06-01

In the Original Investigation titled “Rotating Night Shift Work and Healthy Aging After 24 Years of Follow-up in the Nurses' Health Study,”^1^ published May 4, 2022, the value 46 318 had been incorrectly written as 46 319. This article has been corrected.^1^

## References

[1] Shi H, Huang T, Schernhammer ES, Sun Q, Wang M. Rotating night shift work and healthy aging after 24 years of follow-up in the Nurses' Health Study. JAMA Netw Open. 2022;5(5):e2210450. doi:10.1001/jamanetworkopen.2022.10450 35507343 PMC9069254

