# Supplementary material for: Rotating Night Shift Work and Healthy Aging After 24 Years of Follow-up in the Nurses' Health Study
Source: JAMA Netw Open. 2022 May 4;5(5):e2210450. doi: 10.1001/jamanetworkopen.2022.10450 (PMC9069254; doi:10.1001/jamanetworkopen.2022.10450)
Supplement: Supplement. — eTable 1. Age-Adjusted Baseline Characteristics by Exclusions eTable 2. Mediation of BMI on the Association Between Rotating Night Shift Work and Healthy Aging, NHS (1988-2012) eTable 3. Odds Ratios of Healthy Aging by History of Rotating Night Shift Work in the Nurses’ Health Study (1988-2012), Based on Propensity Weighted Analysis, N = 46,318 eTable 4. Sensitivity Analysis on Rotating Night Shift Work and Healthy Aging in 2012 by Restriction or Adjustment of Additional Potential Confounders, NHS (1988-2012) eTable 5. Stratified Analysis for Rotating Night Shift Work and Healthy Aging in 2012, NHS (1988-2012), N = 46,318 eFigure. Flowchart of Participants’ Selection eMethods 1. Confirmation of Different Chronic Diseases eMethods 2. Assessment of Covariates eMethods 3. Stratified Analysis (Methods and Results) [file jamanetwopen-e2210450-s001.pdf]

Supplementary Online Content

Shi H, Huang T, Schernhammer ES, Sun Q, Wang M. Rotating night shift work and healthy aging after 24 years of follow-up in the Nurses' Health Study. *JAMA Netw Open*. 2022;5(5):e2210450. doi:10.1001/jamanetworkopen.2022.10450

eTable 1. Age-Adjusted Baseline Characteristics by Exclusions

eTable 2. Mediation of BMI on the Association Between Rotating Night Shift Work and Healthy Aging, NHS (1988-2012)

eTable 3. Odds Ratios of Healthy Aging by History of Rotating Night Shift Work in the Nurses' Health Study (1988-2012), Based on Propensity Weighted Analysis, *N*=46,318

eTable 4. Sensitivity Analysis on Rotating Night Shift Work and Healthy Aging in 2012 by Restriction or Adjustment of Additional Potential Confounders, NHS (1988-2012)

eTable 5. Stratified Analysis for Rotating Night Shift Work and Healthy Aging in 2012, NHS (1988-2012), *N*=46,318

eFigure. Flowchart of Participants' Selection

eMethods 1. Confirmation of Different Chronic Diseases

eMethods 2. Assessment of Covariates

eMethods 3. Stratified Analysis (Methods and Results)

This supplementary material has been provided by the authors to give readers additional information about their work.

© 2022 Shi H et al. *JAMA Network Open.*

eTable 1. Age-Adjusted Baseline Characteristics by Exclusions[a]

	Included (*n*=46,318)	Excluded (*n*=12,300)
Age in 1988, years [b]	55.4 (6.1)	56.6 (5.9)
Education, %		
Registered nurse	69.7	71.8
Bachelor	20.1	19.4
Master or doctorate	10.2	8.8
Husbands education, %		
≤ High school	45.6	46.5
College graduate	29.5	29.0
Graduate school	24.9	24.5
Race(white), %	97.8	96.5
Marital status, %		
Married	92.2	92.1
Widowed	3.0	2.7
Separated, divorced, or never married	4.8	5.1
Family annual income, 10,000$	6.0 (4.7-7.7)	6.0 (4.7-7.7)
BMI in 1988, kg/m^2	25.3 (4.6)	25.6 (4.7)
Smoke, %		
Never smoker	44.2	47.5
Past smoker	37.4	36.2
Current smoker	18.4	16.3
Alcohol intake in 1986, %		
None	39.9	43.0
1-14.9 g/d	47.0	45.8
≥15 g/d	13.1	11.2
AHEI in 1986 (no alcohol)	47.6 (10.8)	47.4 (10.8)
Total Calories, kcal	1756.1 (528.2)	1748.7 (541.3)
Total coffee, cup/d	2.5 (1.0-3.5)	2.5 (1.0-3.3)
Physical activity, MET-h/wk[c]	9.0 (3.4-20.9)	8.6 (3.2-20.4)
Standing time, h/day	4.4 (1.6-7.2)	4.4 (1.6-7.2)
Sitting time, h/day	4.4 (1.1-4.4)	2.2 (1.1-4.4)
Family history of diabetes, %	28.8	29.7
Family history of MI, %	18.6	18.2
Family history of cancer, %	14.4	14.0
History of hypertension, %	16.2	17.7
History of high cholesterol, %	19.2	20.6
Use of multivitamin, %	39.2	40.4
Menopausal status and hormone use, %		
pre-menopausal	21.0	20.3
post and Never used	48.9	50.0
post and Past user	3.0	3.3
post and Current user	27.1	26.3
Regular aspirin, at least 2 tabs/week, %	33.3	34.0
Years of night shift work	1.5 (0-4.0)	1.5 (0-4.0)
Night shift work>5 years, %	18.6	19.5
Sleep duration, h/day	7.0 (1.0)	6.9 (1.0)
Regular snorer, %	9.2	9.7

Abbreviations: BMI: body mass index (calculated as weight in kilograms divided by the square of height in meters); MET: metabolic-equivalent; AHEI: Alternate-Healthy Eating Index.
Values are mean (SD) or median (P_{25}, P_{75}) for continuous variables; percentages for categorical variables, and are standardized to the age distribution of the study population.
[a] Excluded for missing information related to healthy aging in 2012.
[b] Value was not age adjusted.
[c] MET-h/wk = sum of the average time/week spent in each activity × MET value of each activity.

© 2022 Shi H et al. *JAMA Network Open.*

eTable 2 Mediation of BMI on the Association Between Rotating Night Shift Work and Healthy Aging, NHS(1988-2012) [a]

Effect	MV-adjusted OR (95%CI) [b]	*P*	Percentage Mediated
Overweight			42.9
Natural Direct Effect (NDE)	1.13 (0.98, 1.29)	0.08	
Natural Indirect Effect (NIE)	1.09 (1.07, 1.11)	<0.001	
Total Effect (TE)	1.24 (1.07, 1.40)	0.006	
BMI (kg/m^2)			61.6
Natural Direct Effect (NDE)	1.10 (0.95, 1.25)	0.20	
Natural Indirect Effect (NIE)	1.14 (1.12, 1.17)	<0.001	
Total Effect (TE)	1.26 (1.09, 1.43)	0.003	

[a]rotating night shift work ≥10y; healthy aging=0, usual aging=1 (note: this code is different from other tables);
[b]MV-adjusted: same as table 3.

© 2022 Shi H et al. *JAMA Network Open.*

eTable 3. Odds Ratios of Healthy Aging by History of Rotating Night Shift Work in the Nurses' Health Study (1988-2012), Based on Propensity Weighted Analysis, *N*=46,318

Outcome	Rotating night shift work	*n*	HA (%)	MV-based PS weight[a]	MV+BMI-based PS weight[b]
**Healthy aging**					
	0y	18838	1653 (8.8)	1	1
	1-5y	18944	1547 (8.2)	1.01 (0.96-1.07)	1.05 (0.99-1.10)
	6-9y	3152	218 (6.9)	0.85 (0.76-0.95)	0.88 (0.79-0.98)
	≥10y	5384	277 (5.1)	0.61 (0.56-0.68)	0.63 (0.57-0.70)
**Free of main chronic diseases**					
	0y	18838	7565 (40.2)	1	1
	1-5y	18944	7528 (39.7)	1.07 (1.04-1.10)	1.09 (1.05-1.12)
	6-9y	3152	1063 (33.7)	0.82 (0.77-0.87)	0.84 (0.79-0.89)
	≥10y	5384	1630 (30.3)	0.70 (0.67-0.74)	0.71 (0.68-0.75)
**Good physical function**					
	0y	18838	3135 (16.6)	1	1
	1-5y	18944	2996 (15.8)	1.03 (0.99-1.07)	1.06 (1.02-1.11)
	6-9y	3152	431 (13.7)	0.88 (0.81-0.95)	0.90 (0.83-0.98)
	≥10y	5384	588 (10.9)	0.67 (0.63-0.72)	0.69 (0.65-0.74)
**Good mental health**					
	0y	18838	8328 (44.2)	1	1
	1-5y	18944	8255 (43.6)	1.06 (1.03-1.09)	1.07 (1.04-1.10)
	6-9y	3152	1228 (39.0)	0.88 (0.83-0.93)	0.89 (0.84-0.94)
	≥10y	5384	1843 (34.2)	0.72 (0.69-0.75)	0.73 (0.69-0.76)
**Good memory function**					
	0y	18838	9748 (51.8)	1	1
	1-5y	18944	9628 (50.8)	1.04 (1.01-1.07)	1.05 (1.02-1.08)
	6-9y	3152	1468 (46.6)	0.88 (0.83-0.93)	0.88 (0.84-0.94)
	≥10y	5384	2325 (43.2)	0.77 (0.74-0.81)	0.77 (0.74-0.81)

Abbreviations: HA: healthy aging;
[a] **MV-based PS weight:** propensity weight was based on: age at baseline (continuous), education (registered nurse, bachelor, or graduate), marital status (married, widowed, or separated/divorced), household income (quintiles); baseline hypertension and high cholesterol (yes or no); family history of cancer, MI and diabetes (yes or no); menopausal status and hormone use (premenopausal, postmenopausal never users, postmenopausal past users, postmenopausal current users), aspirin use (regular use or not); lifestyle factors including smoking history (never, former smoker, current smoker), alcohol intake (none, 1-14.9, ≥15 g/d), total energy intake (quintiles), diet quality (Alternate Healthy Eating Index score, in quintiles), physical activity (metabolic equivalent task-hours per week, in quintiles), standing and sitting time (in quintiles); sleep duration (≤5, 6, 7, 8, ≥9h);
[b] **MV+BMI-based PS weight:** propensity weight was additionally based on: body mass index at baseline (BMI <18.5, 18.5-24.9, 25-29.9, ≥30 kg/m^2).

© 2022 Shi H et al. *JAMA Network Open.*

eTable 4. Sensitivity Analysis on Rotating Night Shift Work and Healthy Aging in 2012 by Restriction or Adjustment of Additional Potential Confounders, NHS (1988-2012)

**Shift work, years**	**adjusted OR (95%CI)**			
	**0**	**1-5y**	**6-9y**	**≥10y**
**Restriction:**				
participants with no hypertension, *n*=38,912	1 (ref)	0.94 (0.87-1.02)	0.90 (0.77-1.06)	0.79 (0.68-0.91)
participants with no hypercholesterolemia, *n*=37,514	1 (ref)	0.95 (0.87-1.03)	0.89 (0.75-1.05)	0.82 (0.71-0.95)
**Adjustment for:**				
MV[a]+ snore, *n*=41,084	1 (ref)	0.95 (0.88-1.03)	0.94 (0.80-1.11)	0.82 (0.71-0.95)
MV[a]+ coffee, *n*=46,318	1 (ref)	0.96 (0.89-1.03)	0.92 (0.79-1.07)	0.79 (0.69-0.91)
MV[a]- sleep duration, *n*=46,318	1 (ref)	0.96 (0.89-1.03)	0.92 (0.79-1.07)	0.79 (0.69-0.91)
MV[a]+WHRq5, *n*=46,318	1 (ref)	0.96 (0.89-1.04)	0.93 (0.80-1.09)	0.82 (0.71-0.94)

[a]MV: same as table 3; WHRq5: quintiles of waist-hip ratio.

© 2022 Shi H et al. *JAMA Network Open.*

eTable 5. Stratified Analysis for Rotating Night Shift Work and Healthy Aging in 2012, NHS (1988-2012), *N*=46,318

Shift work, years	MV-adjusted odds ratio (95%CI)[a]				*P* for interaction
	0	1-5y	6-9y	≥10y	
**Age in 1988** (Attained age in 2012), years					0.10
46-55(70-79) (*n*=24,342)	1 (reference)	0.98 (0.90-1.07)	0.92 (0.78-1.09)	0.84 (0.73-0.98)	
56-68(80-92) (*n*=21,976)	1 (reference)	0.82 (0.67-1.00)	0.83 (0.57-1.21)	0.57 (0.40-0.82)	
**BMI in 1988, kg/m^2**					0.09
<25 (*n*=26,106)	1 (reference)	0.93 (0.85-1.02)	0.88 (0.73-1.06)	0.88 (0.75-1.04)	
≥25 (*n*=20,212)	1 (reference)	1.09 (0.93-1.28)	1.23 (0.92-1.63)	0.84 (0.64-1.09)	
**Physical activity, MET-h/wk**					0.02
<7.5 (*n*=20,040)	1 (reference)	0.85 (0.74-0.97)	0.94 (0.72-1.22)	0.63 (0.49-0.82)	
≥7.5 (*n*=26,278)	1 (reference)	1.02 (0.93-1.13)	0.92 (0.76-1.12)	0.90 (0.76-1.06)	
**Smoking**					0.11
Never smoker (*n*=20,445)	1 (reference)	0.97 (0.87-1.08)	0.73 (0.57-0.93)	0.70 (0.57-0.86)	
Past smoker (*n*=17,345)	1 (reference)	0.94 (0.83-1.06)	1.11 (0.88-1.40)	0.88 (0.70-1.10)	
Current smoker (*n*=8,528)	1 (reference)	0.95 (0.73-1.22)	1.11 (0.73-1.69)	0.86 (0.60-1.25)	
**Alcohol intake, g/d**					0.60
None (*n*=17,543)	1 (reference)	0.97 (0.85-1.11)	1.00 (0.77-1.29)	0.82 (0.66-1.03)	
1-14.9 (*n*=20,692)	1 (reference)	0.96 (0.86-1.06)	0.78 (0.62-0.99)	0.71 (0.57-0.87)	
≥15 (*n*=5,756)	1 (reference)	0.96 (0.76-1.22)	1.15 (0.73-1.81)	1.13 (0.73-1.76)	
**Regular snoring**					0.07
Yes (*n*=3,763)	1 (reference)	0.96 (0.88-1.04)	0.96 (0.81-1.13)	0.86 (0.74-0.99)	
No (*n*=37,321)	1 (reference)	0.90 (0.61-1.32)	0.77 (0.36-1.64)	0.32 (0.14-0.72)	
**Sleep duration, hours**					0.71
≤6 (*n*=11,989)	1 (reference)	1.03 (0.87-1.20)	0.88 (0.65-1.19)	0.90 (0.71-1.13)	
7 (*n*=17,519)	1 (reference)	0.95 (0.84-1.07)	1.04 (0.82-1.31)	0.76 (0.60-0.97)	
≥8 (*n*=11,620)	1 (reference)	0.90 (0.77-1.06)	0.81 (0.57-1.16)	0.77 (0.56-1.06)	
**Lifestyle score**[b]					0.78
0-1 (*n*=11,859)	1 (reference)	0.94 (0.77-1.16)	1.14 (0.79-1.65)	0.70 (0.48-1.01)	
2-3 (*n*=24,751)	1 (reference)	1.00 (0.91-1.11)	0.97 (0.79-1.18)	0.80 (0.67-0.96)	
4-5 (*n*=5,280)	1 (reference)	0.91 (0.76-1.09)	0.66 (0.43-1.02)	0.99 (0.71-1.38)	

[a]MV-adjusted: same as table 3.
[b]Lifestyle score included five lifestyle factors: diet quality, smoking, physical activity, alcohol consumption, and BMI. We defined a healthy diet as an AHEI score in the top 40% of our cohort. For smoking, we defined the low-risk as never smoking. We classified low-risk of physical activity as more than 30 minutes a day of moderate or vigorous activities (including brisk walking) that require at least the expenditure of 3 metabolic equivalents (METs) or more per hour. We defined low-risk alcohol consumption as moderate alcohol consumption, i.e. 5-15 g/day. Low-risk body weight was defined as BMI in the range of 18.5-24.9 kg/m^2. For each factor, the participant received a score of 1 if she met the criterion for low-risk and 0 otherwise (high-risk factor). The sum of these five scores provided a final low-risk score of 0, 1, 2, 3, 4 or 5 with higher scores indicating a healthier lifestyle. Adjusted for same confounders as the main analysis except for lifestyle factors, i.e., age at baseline (continuous), education (registered nurse, bachelor, or graduate), marital status (married, widowed, or separated/divorced), household income (quintiles); baseline hypertension and high cholesterol (yes or no); family history of cancer, MI and diabetes (yes or no); menopausal status and hormone use (premenopausal, postmenopausal never users, postmenopausal past users, postmenopausal current users), aspirin use (regular use or not); sleep duration (≤5, 6, 7, 8, ≥9h).

© 2022 Shi H et al. *JAMA Network Open.*

Flowchart of Participants' Selection

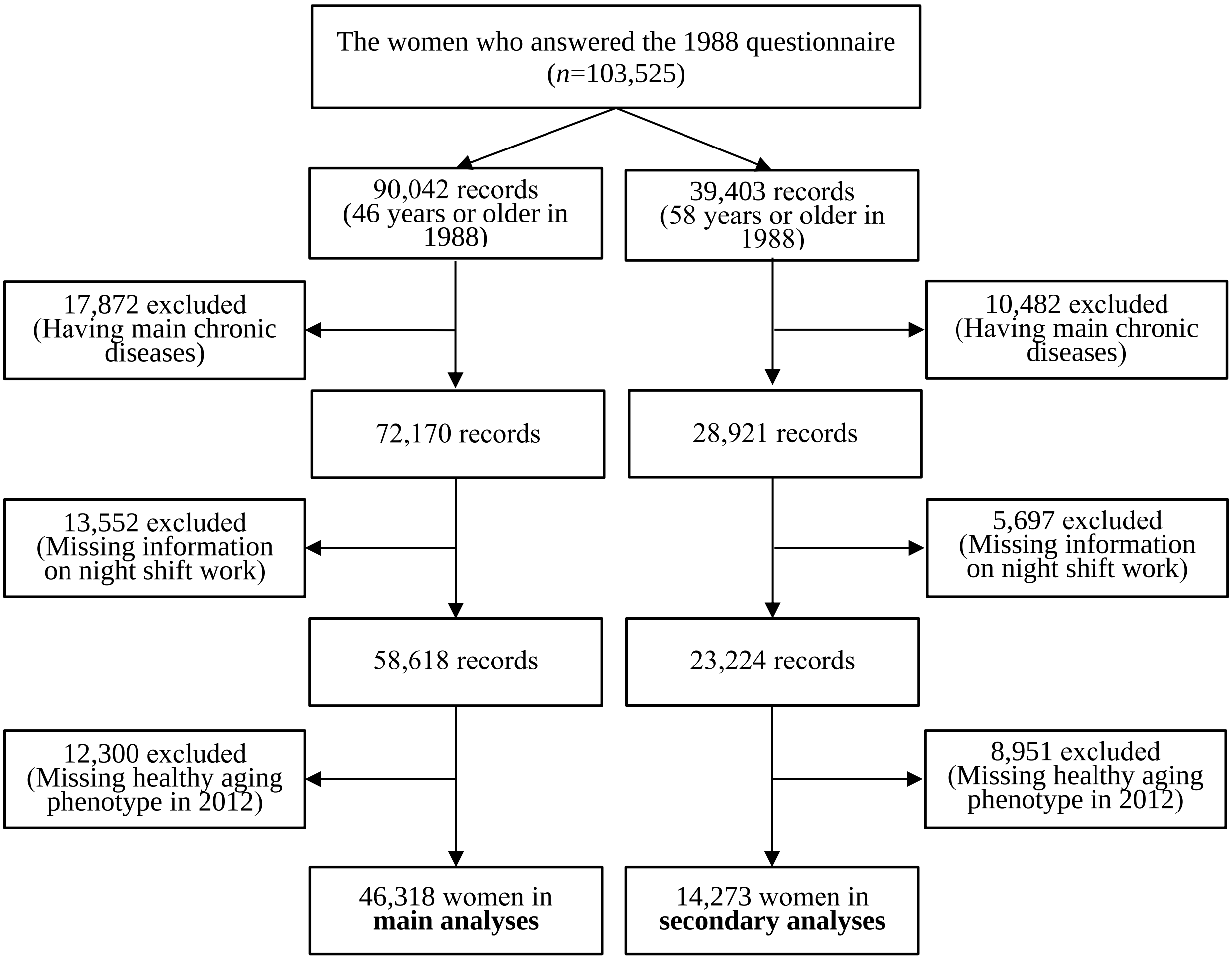

eFigure. Flowchart of Participants' Selection

Main analysis: Of 90,042 women who answered the 1988 questionnaire and were 46 years or older in 1988 (i.e. could reach to age 70 years in 2012), we excluded those who had any of 11 main chronic diseases at baseline (*n*=17,872), had missing information on rotating night shift work (*n*=13,552), or had missing data on healthy aging phenotype in 2012 (*n*=12,300), leaving 46,318 women (age range: 46–68 years) in our main analyses.

Secondary analysis: Of 39,403 women who answered the 1988 questionnaire and those who were 58 years or older in 1988 (i.e. could not reach to age 70 years in 2000), and who had no main chronic diseases at baseline (*n*=10,482), we excluded those who had missing information on rotating night shift work (*n*=5,697), or had missing data on healthy aging phenotype in 2012 (*n*=8,951), leaving 14,273 women (age range: 46–68 years) in our secondary analyses.

© 2022 Shi H et al. *JAMA Network Open.*

eMethods 1. Confirmation of Different Chronic Diseases

Self-reported disease was confirmed by professional staff through medical record or pathology report review, telephone interview, or supplementary questionnaire inquiries, and has been shown valid in this cohort.

Diabetes was confirmed using a validated supplementary questionnaire. Myocardial infarction and stroke were adjudicated using medical records, so were cancer, Parkinson disease, and multiple sclerosis. Coronary artery bypass graft surgery, congestive heart failure, kidney failure, amyotrophic lateral sclerosis, and chronic obstructive pulmonary disease (COPD) are based on self-reports.

© 2022 Shi H et al. *JAMA Network Open.*

eMethods 2. Assessment of Covariates

Information on a broad range of covariates were obtained, including demographic characteristics such as marital status, race, education, and household income; lifestyle factors (physical activity, sleep behavior, and dietary data); family history of cancer, myocardial infarction, and diabetes; clinical diagnoses of hypertension and high cholesterol; use of supplemental multivitamin and aspirin; menopausal status and postmenopausal hormone use.

Median annual household income and home value were estimated from the census tract of participant's residence, geocoded to the 1994 U.S. Census. Weight and height were collected on the 1976 questionnaire, and weight was further requested every two years thereafter. Self-reported weight was highly correlated (r=0.96) with measured weight in a previous validation study.[40]

Dietary data were collected using a validated semi-quantitative food frequency questionnaire,[41-43] based on which we derived total energy intake, alcohol and coffee consumption, and the Alternate Healthy Eating Index (AHEI). Women reported the average total hours of sleep in 24 hours and frequency of snoring in 1986. Specifically, we defined regular snorers as those who reported snoring regularly, and non-snorers as those who only occasionally or almost never snored.

Physical activity was assessed based on participants' reports regarding the following recreational activities: walking, jogging (>10 minutes/mile), running (≤10 minutes/mile), bicycling, tennis/squash/ racquetball, swimming, calisthenics/ aerobics/ aerobic dance/rowing machine, yoga/stretching/toning, and lawn mowing. Based on this information and intensity of each physical activity as expressed by the metabolic equivalent of task (MET), total weekly MET-hours were calculated. Participants were dichotomized into being physically active and inactive, using a threshold of 7.5 MET-h/wk, which corresponds to the minimum current physical activity recommendations.[44] The reproducibility and validity of this questionnaire in our cohorts has been demonstrated previously.[45,46]

© 2022 Shi H et al. *JAMA Network Open.*

eMethods 3. Stratified Analysis (Methods and Results)

In the primary outcome analysis, we conducted stratified analysis by age in 1988 (46-55, ≥56 years), BMI (<25, ≥25 kg/m^2), physical activity (<7.5, ≥7.5 MET-hours/wk), smoking (never, former smoker, current smoker), alcohol consumption (none, 1-14.9, ≥15 g/d), regular snoring (yes, no) and sleep duration (≤6, 7, ≥8). In order to evaluate whether an overall healthy lifestyle can attenuate the adverse effects of rotating night shifts on healthy aging, we also conducted a stratified analysis according to a lifestyle score based on five factors,[51] including a high diet quality score (upper 40%), never smoking, ≥30 min/d of moderate to vigorous physical activity, moderate alcohol consumption, and BMI of 18.5 to 24.9 kg/m^2. Statistical interactions were assessed by likelihood ratio tests comparing the models with and without the multiplicative interaction terms.

Stratified analysis (results)

Overall, we did not observe statistically significant differences in the association by several pre-specified factors ($P_{for\ interaction}$ >0.05; **eTable5**), except for physical activity. The inverse association of rotating night shift work with healthy aging was statistically significant among physically inactive women and no association was observed for physically active women ($P_{for\ interaction}$=0.02). Moreover, the inverse association between rotating night shift work and healthy aging was more evident among older women although $P_{for\ interaction}$ =0.10: among those aged 46-55 years at baseline (survived to 70-79 years in 2012), the MV-adjusted OR comparing those with ≥10 years vs. those with no rotating night shift work was 0.84 (95% CI: 0.73 to 0.98); by contrast, among participants aged 56-68 years at baseline (survived to 80-92 years in 2012), the corresponding OR was 0.57 (95% CI: 0.40 to 0.82).

© 2022 Shi H et al. *JAMA Network Open.*
